# Bite Force Transducers and Measurement Devices

**DOI:** 10.3389/fbioe.2021.665081

**Published:** 2021-04-09

**Authors:** Yingzhi Gu, Yuxing Bai, Xianju Xie

**Affiliations:** Department of Orthodontics, Beijing Stomatological Hospital, Capital Medical University, Beijing, China

**Keywords:** bite force, force transducer, masticatory system, bite force device, force measurement

## Abstract

In dental research, bite force has become an important curative effect evaluation index for tooth restoration, periodontal treatment, and orthodontic treatment. Bite force is an important parameter to evaluate the efficacy of the masticatory system. Physicians obtain the therapeutic basis for occlusal adjustment by measuring the bite force and the dynamic changes in occlusal contact at different stages of treatment and objectively evaluate the therapeutic effect. At present, many devices are used to record the bite force. Most of these devices use force transducers to detect bite force, such as strain gauge transducers, piezoresistive transducers, piezoelectric transducers, optical fiber transducers, and pressure-sensitive films. This article summarizes the various equipment used to record bite force, related materials and the characteristics of this equipment. It provides a reference for physicians to make choices during the clinical process and at the same time provides a basis for the development of new occlusal force measurement materials.

## Introduction

The function and integrity of the masticatory system have an important impact on a person’s quality of life (Fujimoto et al., 2020). Poor health of the masticatory system may be caused by many interrelated factors, including tooth decay, tooth loss, malocclusion, temporomandibular joint dysfunction, and mandibular fractures. Therefore, timely diagnosis and treatment of these diseases are essential to improve the quality of life (Moghadam et al., 2020). Bite force can be defined as “the force exerted by the masticatory muscles upon the occlusal surfaces of teeth,” and the maximum bite force of the natural teeth of healthy adults in the molar area is between 300 and 600 Newtons (N) (Bakke, 2006). Decreased bite force may be related to periodontal tissue loss, trauma and temporomandibular joint disorders (Williams et al., 1987; Pereira et al., 2007a), and increased bite force may be related to bruxism (Gibbs et al., 1986).

The research on bite force has a long history. In 1681, Borelli first studied the bite forces and designed a gnathodynamometer. After that, several researchers continued to develop devices to measure bite force, including lever-spring, monometerspring and micrometered instruments (Ortuğ, 2002). Today, various sensitive electronic devices are in use to measure bite force. These devices use pressure sensors to convert force into electrical energy and can be divided into strain gauge transducers, piezoelectric transducers, piezoresistive transducers, and pressure transducers (Collins, 2015). There are already many commercially available devices used to record bite force, such as the Dentoforce 2 (ITLAB, Sollentuna, Sweden), IDDK (Kratos, Cotia, São Paulo, Brazil), FSR No. 151 (Interlink Electronics Inc., Camarillo, CA, United States), Flexiforce (Tekscan, South Boston, MA, United States), GM10 (Nagano Keiki, Japan), MPX 5700 (Motorola, SPS, Austin, TX, United States), T Scan system (Tekscan, Inc., South Boston, MA, United States) and the Dental Prescale system (GC Co. Ltd., Japan). These devices can be used to assist in the diagnosis of pre-existing temporomandibular disorders, mandibular fractures and malocclusion deformities, and can be used to evaluate the treatment efficacy by comparing the bite force values before and after an intervention (Alam and Alfawzan, 2020; Kruse et al., 2020b).

In this review, we introduce several common clinical pressure transducers for bite force measurement and novel bite force transducers developed in recent years. It provides a reference for physicians to make choices during the clinical process and at the same time provides a basis for the development of new occlusal force measurement materials.

## Transducers Currently Used for Bite Force Measurement

### Strain Gauge Transducer

A strain gauge transducer is a pressure transducer that uses elastic sensitive elements and strain gauges to convert the measured pressure into a corresponding change in resistance value (Jansen van Vuuren et al., 2020). It is made up of resistance strain gauges, elastic elements, and compensation resistors, generally used to measure larger pressures. The core element of the strain gauge transducer is the resistance strain gauge, which is a sensing element that can convert the strain change on the mechanical component into a resistance change. The resistance change of the strain gauge is proportional to the deformation. Therefore, the voltage or current change recorded in the display can be used to determine the strain on the test piece (Kim et al., 2020). Strain gauge transducers have a high sensitivity and accuracy, a large measuring range, a small size, and a lightweight, and can be adapted to use in various environments.

Dentoforce 2 (ITLAB, Sollentuna, Sweden) is a bite force measurement device with a metal bite fork covered with rubber and equipped with a strain gauge sensor, which can be placed in the bite area and allow the subject to bite it. The bite fork is connected to the recorder, and the bite force value applied can be displayed on the digital display device (Multimeter 4055. ITL AB, Solientuna. Sweden) (Verma et al., 2017). The device can not only display instantaneous readings but also display the minimum and maximum forces during the measurement period and it can measure forces up to 1000 N. The thickness of the bite fork is 11 mm. After the bite fork is in place, the subject is asked to bite as hard as possible for 3–4 s (Verma et al., 2017). The equipment has been successfully used for research purposes (Tzakis et al., 1992; Ernberg et al., 1996). Nevertheless, in recent years, there has been little research on this sensor.

IDDK (Kratos, Cotia, São Paulo, Brazil) is a digital dynamometer consisting of a bite fork. The bite fork is made up of two metal rods with a plastic disk as the shell and it is connected to the digital monitor by a flexible cord. The device has a scale in kg or N and a “set to zero” button with a measuring capacity of 1000 N or 100 kg (Vilela et al., 2017). The thickness of the bite fork is 14.6 mm. When measuring the bite force, the bite fork is placed between the teeth, and the subject bites on the plastic plate to record the bite force. When a force is applied, the metal rod will deviate, generating an electric signal, which is transmitted to the digital monitor (Kogawa et al., 2006).

The device has been successfully used in many studies to record bite force. Garcia et al. used IDDK to evaluate the bite force of children with cleft lip and palate. The results of that study showed that the bite force of children with cleft lip and palate was no different from that of normal children, and there was no significant difference between the bite force on the cleft side and the non-cleft side of a unilateral cleft lip and palate (Garcia et al., 2016). Pepato et al. used IDDK to evaluate the occlusal force of mandibular angle fractures and mandibular condyle fractures before and 2 months after surgery and found that the patient’s occlusal force increased significantly after surgery (Pepato et al., 2014). Da Silva et al. used IDDK to evaluate the occlusal force of 16 male and female patients wearing complete dentures and implant overdentures. The study found that the maximum occlusion of the incisors and molars after 15 months of implant overdentures placement in the mandible was significantly increased relative to wearing complete dentures, suggesting that the use of mandibular implant overdentures should be the first choice for patients needing complete dentures (da Silva et al., 2011).

Although the strain gauge transducer has been proven to measure the bite force accurately, it is still difficult to record the true maximum bite force. This was mainly due to the hard surface of the sensor, and the subjects felt uncomfortable or worried when biting it (Soni et al., 2020). Some researchers have tried to use protective covers of different materials (such as acrylic resin and PVC) to cover the metal bite fork surface to make the bite more comfortable, but this does not overcome the fear of biting down on hard surfaces (Fernandes et al., 2003). Another major disadvantage of the strain gauge transducer is that the location of the bite fork may affect the bite force measurement results. Some researchers have pointed out that the change in bite force is related to the position of the sensor relative to the dental arch. The bite force measured by the sensor located further distal is greater than mesial, which may be attributed to the mechanical leverage caused by the thick metal plate of the bite fork used in the strain gauge transducer (Braun et al., 1996).

### Piezoresistive Transducer

A piezoresistive transducer is made by using the piezoresistive effect of a single crystal silicon material and integrated circuit technology. After the monocrystalline silicon material receives the force, the resistivity changes, and the electrical signal output proportional to the force change can be obtained through a measurement circuit (Chen et al., 2019). It has the characteristics of high sensitivity, a fast dynamic response, high measurement accuracy, good stability, wide operating temperature range, a small size, and easy mass production, so it has been widely used. It overcomes the problems of strain gauge transducers and can integrate resistance, compensation circuits, and signal conversion circuits on silicon chips and even integrate calculation processing circuits and sensors together (Song et al., 2020b). It is a promising transducer and is widely used in the medical business. At present, there are various miniature sensors used to measure the pressure in the cardiovascular system, intracranial system, urethra, and eyeballs (Sattayasoonthorn et al., 2019; Song et al., 2020a; Wang et al., 2020).

**FIGURE 1:**
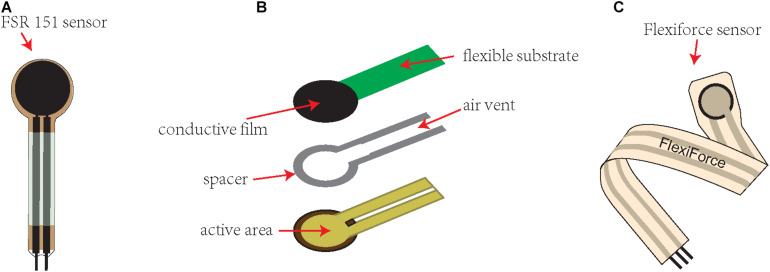
Commonly used piezoresistive sensors for bite force measurement. **(A)** An illustrative figure of the FSR 151 (Interlink Electronics Inc., Camarillo, CA, United States) sensor. **(B)** An illustrative figure showing the construction of the FSR151 sensor, consisting of two thermoplastic plastic sheets. Two conductive electrodes crossing each other are embedded on the bottom sheet, and semiconductive polyetherimide ink is coated on the top sheet. **(C)** An illustrative figure of the Flexiforce (Tekscan, South Boston, MA, United States) sensor.

FSR 151 (Interlink Electronics Inc., Camarillo, CA, United States) is a piezoresistive transducer that measures bite force. It consists of two thermoplastic plastic sheets. Two conductive electrodes crossing each other are embedded on the bottom sheet, and semiconductive polyetherimide ink is coated on the top sheet (Slingsby et al., 2001), as illustrated in Figure 1. The basic feature of the sensor is that it is piezoresistive; that is, its resistance decreases with increasing pressure. The circular sensor has a diameter of 12 mm and a thickness of 0.25 mm. This device has been used in many bite force studies. Goncalves et al. used FSR151 to assess whether hormone levels affect the maximum bite force (MOF) of healthy women at different stages of the menstrual cycle. The maximum bite force on both sides of the molar area was evaluated. It was found that there was no difference in the maximum bite force during the subjects’ menstrual cycle phases (Gonçalves et al., 2011). Bavia et al. divided 48 women into three groups based on craniofacial morphology and used FSR151 to evaluate their maximum bite force. They found that the maximum bite force of each group was significantly different. It is speculated that the craniofacial shape affects the maximum bite force (Bavia et al., 2016).

Flexiforce (Tekscan, South Boston, MA, United States) is a piezoresistive transducer for measuring the bite force of small mammals. It includes a piezoresistive sensor and an electronic device for detecting changes in sensor resistance (Pais Clemente et al., 2019). The maximum force that can be measured is 4500 N. The piezoresistive sensor is a thin plastic strip 10 mm wide, 150 mm long and 0.2 mm thick. The round part of the tip is a piezoresistive material, which acts as a variable resistor. As the applied force increases, its resistance decreases. The electronic device used to measure the resistance change of the sensor is a circuit connected to a B2pe microcontroller (Parallax, Inc., Rocklin, CA, United States) (Freeman and Lemen, 2008). Some scholars have modified the device to improve the contact between the occlusal surface of the tooth and the sensor, partially covering the teeth with a preset resin bite block, and guiding the sensor into the preset depression on the bite block so that the distance between the jaws remains the smallest during occlusion (Kruse et al., 2020a). Piezoresistive sensors are inexpensive, easy to use and have been successfully used in many studies. Clemente et al. used Flexiforce to measure the pressure exerted on the instrument by the incisor of a wind instrument player and believed that this sensor could be used to identify the tooth that exerts the greatest force on the instrument during the player’s performance (Clemente et al., 2018). Valentim et al. used Flexiforce to measure the force exerted by the tongue and upper lip on the teeth of 28 subjects during rest and swallowing and found that the lips exerted more force on the upper central incisor than the tongue does at rest, while during swallowing, there was no difference between the force of the tongue and the lips on the teeth (Valentim et al., 2014). However, some scientists have shown that these sensors are not as accurate as other types of pressure sensors (Athavale et al., 2020; Nandasiri et al., 2020).

### Pressure Transducer

A pressure transducer is a device that converts fluid or gas pressure into electrical signals. It includes a chamber filled with fluid or air. When receiving pressure, the pressure in the chamber increases and is transmitted to the pressure gauge for measurement. Based on the contents of the chamber, pressure transducers can be divided into pneumatic transducers and hydraulic transducers (Peng et al., 2020; Rosier, 2020).

The GM10 (Nagano Keiki, Japan) dynamometer is a hydraulic transducer consisting of a hydraulic gauge and an occlusal element made of vinyl material (Ibraheem and El-sisy, 2020). A maximum bite force of 1000 N can be measured. The main advantage of the GM10 dynamometer is that it is portable, the bite element is soft, and the bite force can be recorded safely and comfortably. The accuracy and repeatability of the dynamometer have been previously confirmed and it has been successfully used in several studies to record the bite force of human dentition. Al-Omiri et al. used GM10 to evaluate the difference between the maximum bite force of a fixed tooth supported by an implant and the maximum bite force of the contralateral denate side and to determine the influence of sex, height and body mass index (BMI) on the maximum bite force. It was found that the maximum bite force of the fixed tooth supported by the implant was lower than the maximum bite force of the natural tooth side. The maximum bite force was higher for men and taller participants. However, there was no significant correlation between BMI and MBF value (Al-Omiri et al., 2014). To determine whether the maximum bite force (MBF) is related to frailty in elderly individuals, Iwasaki et al. conducted a prospective cohort study of 322 75-year-old subjects. Researchers used GM10 to measure the maximum bite force and found that lower MBF indicates poor oral function, which increases the health risks of older men and women (Iwasaki et al., 2018).

Some scientists have also proposed improvements of GM10. Serra et al. replaced the original hard occlusal surface of GM10 with a soft occlusal surface and measured the subject’s maximum voluntary bite force. They found that the measured force value on the soft occlusal surface was greater; therefore, it is recommended to use a soft occlusal surface in a maximum voluntary bite force record to improve its reliability (Serra and Manns, 2013).

MPX5700 (Motorola, SPS, Austin, TX, United States) is a pneumatic pressure transducer. In this system, pipes and sensors are connected to an analog-to-digital converter. The system has developed software for reading pressure signals, and the read pressure data are connected to a computer using Excel to generate text files (Pereira et al., 2007b). Because the tube is flexible, the subject can easily adapt to it. In addition, the tube elastically deforms during occlusion, which conforms to the occlusal anatomy of a single upper and lower teeth, thereby making the force distribution more uniform (Pereira et al., 2007b). However, the MPX 5700 pressure sensor media is only suitable for use with air. Except for dry air, any other pressure medium may adversely affect the sensor performance and long-term reliability (Serra et al., 2007).

### Piezoelectric Transducer

A piezoelectric transducer is a kind of transducer that uses the piezoelectric effect of a piezoelectric material to convert the measured pressure into an electric signal. Its sensitive element is made of piezoelectric material (Bing et al., 2020). Piezoelectric materials in piezoelectric sensors generally include piezoelectric crystals (i.e., quartz crystals), piezoelectric ceramics, and polymer piezoelectric materials (González et al., 2016). Piezoelectric transducers have a small size and a light weight. Because they have no moving parts, they have a solid structure, good reliability, and high stability.

The piezoelectric material generates electric charges on the surface after being forced, and after the charge amplifier and the measuring circuit amplify and transform the impedance, it becomes an electrical output proportional to the external force. Quartz crystals are the earliest applied piezoelectric material. With the large-scale application of piezoelectric sensors, many artificial crystals have been developed using quartz, such as piezoelectric single crystals (Zhu et al., 2020). However, due to their performance defects, these artificial single crystals have gradually been replaced.

Currently, piezoelectric ceramics are mostly used as materials for piezoelectric sensors. Piezoelectric ceramics use the electrostrictive effect of polycrystalline piezoelectric ceramics. The current most commonly used piezoelectric ceramic is lead zirconate titanate (PZT) (Liu et al., 2020). Both piezoelectric single crystals and piezoelectric ceramics are brittle materials. The piezoelectric polymer film represented by polyvinylidene fluoride (PVDF) has strong piezoelectricity and flexibility; in particular, the acoustic impedance is close to that of water and biological tissues and is a good material for making sensors (Abdolmaleki and Agarwala, 2020). Piezoelectric composite materials of piezoelectric ceramics and polymers have also been used in the field of piezoelectric sensors (Oh et al., 2020).

**FIGURE 2:**
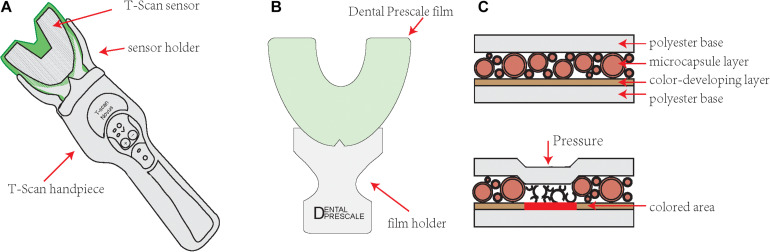
Illustrative figure of the T Scan system (Tekscan, Inc., South Boston, MA, United States) sensor and Dental Prescale system film. **(A)** A T-scan sensor is shaped to fit the dental arch. There are control buttons on the handpiece, which is convenient for doctors to operate. **(B)** A dental prescale system film is shaped to fit the dental arch. **(C)** The dental prescale system film is comprised of two polyethylene terephthalate films and many microcapsules containing color-forming materials between them. When the bite force is applied, the microcapsules collapse, and the color former contained in the capsule leaks out to react with the developer and form a red color.

The T Scan system (Tekscan, Inc., South Boston, MA, United States) is a set of precision instruments developed by the American Tekscan company that can accurately record the occlusal contact time, force, and area and dynamically analyze the occlusal contact conditions. It consists of a piezoelectric foil sensor (Assery et al., 2020). The first-generation sensor (G1) consists of a polyester film laminated pressure-sensitive ink grid with a dental arch shape (as shown in Figure 2). When it is placed in a mouth and a load is applied, the sensor relays the real-time bite contact sequence and relative force information to the computer software. The new generation sensor T-scan III uses a 0.004-inch, 0.1 mm ultrathin and flexible bite sensor to record the force. The sensor is shaped to fit the dental arch, and the patient’s occlusal process is accurately and quantitatively determined by computer analysis software. At the same time, a three-dimensional map of the dynamic change in the patient’s occlusal force can be obtained, and the patient’s abnormal occlusal force distribution points and occlusal contact area can be accurately marked (Dergin, 2018). In this way, dentists can analyze and judge the occlusal relationship more intuitively and it will not hinder the subject’s jaw movement during the recording process. However, some scholars believe that due to the insufficient flexibility of the bite sensor, the bite force cannot be accurately measured (Heuser et al., 2020). In addition, the range of bite force measured by the device is narrow, and the sensitivity and planar resolution capacity of the device are also insufficient (Koos et al., 2010).

### Pressure Sensitive Film

Japan Fuji Co., Ltd. launched the Dental Prescale system in the early 1990s. The prescale system is a pressure-sensitive film (Dental Prescale; Fujifilm Co., Tokyo, Japan) with analysis equipment (Occluzer FPD703; GC Corp., Tokyo, Japan) (Shiga et al., 2020). The pressure-sensitive film is a pressure-sensitive horseshoe-shaped sheet comprised of two polyethylene terephthalate films and many microcapsules containing color-forming materials placed between them. When a bite force is applied, the microcapsules collapse, and the color former contained in the capsule leaks out to react with the developer and form a red color (Oueis, 2009), as illustrated in Figure 2. According to the pressure applied, different color densities are formed. As the pressure increases, the red becomes more intense.

After recording, the film is stored in a light-resistant container and transported at room temperature for analysis. To maintain the reliability of the measurement, the data must be analyzed on the same day using the analysis equipment Occluzer FPD705 (FujiFilm GC) (Shiga et al., 2020). By scanning the colored image after compression, the number, position, force size and force distribution of the occlusal contact points are obtained.

The main advantages of the Dental Prescale system are the ability to measure the occlusal force and occlusal contact area close to the position between the teeth, and it will not interfere with the occlusion when measuring the occlusal force. However, this system cannot perform continuous measurements. In addition, the pressure-sensitive film needs to be further analyzed by analytical equipment, which is time-consuming.

The Dental Prescale system is currently mainly used in research on oral prosthetic occlusal contact analysis, occlusal force evaluation after maxillofacial surgery, and related influencing factors of temporomandibular joint disease. Hasan et al. used the Dental Prescale system to measure the bite force of edentulous patients with dentures and patients who received implant-supported dentures. Regardless of which implant system is used, the bite force after implantation of the implant will be improved. However, the degree of improvement is obviously related to the original bone quality of the mandible at the implant insertion area (Hasan et al., 2016). Choi et al. used the Dental Prescale system to evaluate the longitudinal changes in the occlusal force and occlusal contact area after intraoral vertical bronchus osteotomy (IVRO). The results showed that the bite force and bite contact area gradually increased throughout the postoperative evaluation period. Increasing the occlusal contact area may be necessary to improve the bite force after surgery (Choi et al., 2014). Gokcen-Rohlig et al. used the Dental Prescale system to evaluate the effect of low-level laser treatment on the occlusal contact area, occlusal pressure, and occlusal force of patients with temporomandibular joint disease. This also indicated that the jaw movement of all patients was significantly improved after laser irradiation, and the pain caused by palpation was also significantly reduced. However, no significant changes were found in the maximum bite force, bite contact area or bite pressure after treatment, and the value after treatment was still significantly lower than that of healthy individuals (Gökçen-Röhlig et al., 2013). Compared with the T-scan bite analysis system, the Dental Prescale system is still an emerging technology in the field of oral research. Its current applications in the field of bite force research needs further exploration and promotion.

The characteristics of the above transducers are listed in Table 1.

**TABLE 1:** Transducers currently used for bite force measurement.

Type	Strain gauge transducer		Piezoresistive transducer		Pressure transducer	Piezoelectric transducer	Pressure sensitive film
Product	Dentoforce2	IDDK	FSR151	Flexiforce	GM10	T scan	Dental Prescale
Company	ITLAB, Sollentuna, Sweden	Kratos, Cotia, São Paulo, Brazil	Interlink Electronics Inc., Camarillo, CA, United States	Tekscan, South Boston, MA, United States	Nagano Keiki, Japan	Tekscan, Inc., South Boston, MA, United States	Fujifilm Co., Tokyo, Japan
Composition	metal bite fork covered with rubber, strain gauge sensor	Metal bite fork, covered with plastic disk, digital monitor	thermoplastic plastic sheets embedded with conductive electrodes and semi-conductive polyetherimide ink	piezoresistive sensor, electronic decting device	hydraulic gauge, vinyl occlusal element	piezoelectric foil sensor	pressure sensitive film, analysis equipment
Advantage	high sensitivity and accuracy, large measuring range, small size, light weight, and can adapt to various environments.		high sensitivity, thin, light weight, cheap, the circular sensor has a diameter of 12 mm and a thickness of 0.25 mm	high sensitivity, thin, light weight, cheap, the sensor is a thin plastic strip 10 mm wide, 150 mm long and 0.2 mm thick.	portable, the bite element is soft, and the bite force can be recorded safely and comfortably.	0.1 mm ultra-thin and flexible bite sensor, occlusal process can be accurately and quantitatively determined.	will not interfere with the occlusion when measuring the occlusal force
Limitations	The thickness of the bite fork is more than 10 mm. interfere with the occlusion when measuring the occlusal force		Less accurate than strain gauge transducer		less reliable.	insufficient flexibility of the bite sensor, narrow range, insufficient sensitivity.	cannot perform continuous measurement, needs to be analyzed by analytical equipment.

## Newly Developed Devices for Bite Force Measurement

In addition to commercially available bite force measurement devices, many scientists have developed new bite force measurement devices in recent years. Lin et al. developed a novel flexible force sensor array to measure the force distribution on the first molar. The developed force sensor array is comprised of flexible polyimide electrodes and barium titanate-based multilayer ceramic capacitors (MLCCs). The piezoelectric and material properties of industrial-grade MLCCs are very suitable for measuring large loads (Lin et al., 2011). The sensor is cheap and easy to integrate with automated manufacturing processes. Before conducting experimental measurements, the author systematically measured and evaluated the force response of the MLCC sensor units, thus confirming their high fracture strength and good sensing performance. The finite element (FE) simulation results show that the sensor has high sensitivity and linearity under a high-speed cycle load of 500 N/s that simulates normal chewing. The error of the total force measured in the artificial tooth using the developed sensor array is less than 5%. Therefore, the developed flexible force sensor array has good potential for low-cost and reliable bite force measurement.

Lantada et al. proposed a new system for measuring human bite force. It is comprised of a passive force sensor located in the oral splint and an active external unit that energizes the sensor and permanently records all force measurements. They can remotely record and process information about the patient’s tooth activity without placing a battery in the mouth (Lantada et al., 2012). The passive force sensor in the mouth consists of two different subsystems: the first subsystem is a power subsystem, consisting of a tuned LC circuit, a Schottky diode rectifier, a low power, and a low dropout voltage regulator (such as MC78 LC30). The regulator provides two capacitors at its input and output terminals to provide a constant DC voltage. This method avoids placing a battery in the mouth. The second subsystem is a force-sensitive oscillator, which consists of a low-power relaxation oscillator (LMC555 from National Semiconductor) and some passive components. The system can perform permanent bite force measurements.

Takahashi et al. developed a metal-free bite force meter, which is designed to omit any electronic or metal components so that it can operate safely in a magnetic field. The device contains a micropressure sensor made of optical fibers (FOP-M-BA; Fiso Technologies Inc., Quebec, QC, Canada) and plastic parts (water bag, pipe and check valve) (Takahashi et al., 2016). Heat-sealable plastic sheets were used to make water bags to match the dental arch of each patient. By reducing the length of the water bag, bites between the upper and lower molars can be avoided. After filling the bag with water and using a sensor (EVO-SD-2; Fiso Technologies, Inc.) the device can measure the change in the internal water pressure. The force applied to each bag was used to monitor the generated pressure using a dynamometer (ZP-1000N; IMADA Co., Ltd., Toyohashi, Japan) to obtain a linear calibration curve for each bag. Therefore, after calibration, the internal water pressure can reflect the bite force applied.

Umesh et al. developed a method to dynamically measure the bite force generated by a single tooth using a fiber Bragg grating bite force recorder (FBGBFR). The proposed FBGBFR is an intraoral device designed to convert the bite force applied on the occlusal surface into strain changes on the substrate and then sense it through the FBG sensor above it (Umesh et al., 2016). The developed device consists of two rectangular rods with dimensions of 100 mm × 5 mm × 4 mm, which are riveted in the center by a movable joint, which makes the two rods imitate the action of scissors. The fiber Bragg grating sensor is glued on a rectangular plate, and the sensor can acquire strain changes on it. The rubber film is attached to the occlusal platform to provide cushioning for the teeth while applying an occlusal force. The magnitude of the strain change on the rectangular plate directly depends on the magnitude of the force exerted on the occlusal platform. The bite force measuring device converts the applied bite force into a strain change, and the strain change is acquired by a fiber Bragg grating sensor.

Fastier et al. developed a low-cost, reliable maximum voluntary bite force sensor. The sensor consists of two polyvinylsiloxane (PVS) silicone layers, an acrylic frame and a metal strain gauge (Fastier-Wooller et al., 2016). The PVS silicone resin acts as a protective layer to reduce the pain that may be caused to the subject when biting on the sensor. A strain gauge attached to the inside of the acrylic resin serves as a sensing element, and an acrylic frame is used to transmit the mechanical strain caused by the bite force to the strain gauge. The sensor is designed for easy manufacturing, assembly, calibration and safe use. It can be used within 1 h of starting production, allowing rapid prototyping and modification. The measured data show a good linear relationship between the applied force and the sensor resistance.

Jansen et al. developed a bite force sensor that can measure the maximum voluntary bite force between opposite tooth surfaces. The sensor frame is composed of two Sandvik 12c27 martensitic stainless steel plates (90 mm × 5 mm × 2 mm), and both ends are supported by a bridge structure. The bridge span in the occlusal recording area is 28 mm. All components are fixed together with high-torque 1.1 Nm high-strength steel bolts. The sensor is a resistance sensor, and each sensor is a strain-dependent thin-film resistor matched with 120-ohm resistance. The author also used the sensor to measure the bite force of forty people to verify the function and clinical application of the device.

Kurosawa et al. developed a headset-type wearable device to indirectly measure bite force by measuring ear canal movement through ear sensors (Kurosawa et al., 2019). Chewing causes changes in the shape of the masticatory muscles and the ear canal near the temporomandibular joint. The ear sensor optically and non-invasively measures this shape change of the ear canal during obstruction. The small photoelectric sensor is connected to the ear sensor. The photoelectric sensor is equipped with a light emitting diode (LED) with an emission wavelength of 940 nm and a phototransistor. The ear sensor irradiates the skin of the ear canal with infrared light, and then the reflected light is received by a phototransistor to measure changes in the shape of the ear canal. The author simultaneously measured six ear canal movements (ear sensor values) from five subjects, the surface electromyogram (EMG) of the masseter muscle, and the bite force as basic research during the development of the bite force meter. Then, these results were used to study the correlation coefficient between the ear sensor value and the bite force. The results showed that there was a strong correlation between the ear sensor value and the bite force. Using this method, the bite force can be measured indirectly.

## Conclusion

The measurement of bite force is of great significance in stomatology, and careful selection of the measurement method and the measuring device are the keys to accurate measurement. Strain-gauge transducers have high sensitivity and good repeatability. However, due to its thickness, it interferes with normal occlusion. Flexiforce and T-scan are both made of thin film, which does not interfere with the occlusion when measuring the bite force. However, after repeated use of the T-scan film, its accuracy will be reduced, so its repeatability is questionable. Therefore, the future development and design of bite force transducers should focus on reducing the thickness of the sensor as much as possible, improving the anti-tear and anti-bite ability of the sensor, and improving the accuracy of the sensor. The development of new materials provides a research foundation for the development of bite force sensors. At the same time, bite force equipment is gradually being miniaturized. It is believed that in the near future, bite force measurement devices will continue to be developed to meet the increasing clinical needs and to provide more convenience for patient diagnosis and treatment.

## Author Contributions

YG conceived the original idea. YG wrote the manuscript with support from XX and YB. All authors contributed to the article and approved the submitted version.

## Conflict of Interest

The authors declare that the research was conducted in the absence of any commercial or financial relationships that could be construed as a potential conflict of interest.
